# Socio-ecological conditions and female infidelity in the Seychelles warbler

**DOI:** 10.1093/beheco/arz072

**Published:** 2019-05-30

**Authors:** Sara Raj Pant, Jan Komdeur, Terry A Burke, Hannah L Dugdale, David S Richardson

**Affiliations:** 1 Centre for Ecology, Evolution and Conservation, School of Biological Sciences, University of East Anglia, Norwich Research Park, Norwich, UK; 2 Groningen Institute for Evolutionary Life Sciences, Faculty of Science and Engineering, University of Groningen, Groningen, The Netherlands; 3 Department of Animal and Plant Sciences, University of Sheffield, Sheffield, UK; 4 School of Biology, Faculty of Biological Sciences, University of Leeds, Leeds, UK; 5 Nature Seychelles, Roche Caiman, Mahe, Republic of Seychelles

**Keywords:** cooperative breeders, extra-pair paternity, group size, infidelity, relatedness, socio-ecological conditions

## Abstract

Within socially monogamous breeding systems, levels of extra-pair paternity can vary not only between species, populations, and individuals, but also across time. Uncovering how different extrinsic conditions (ecological, demographic, and social) influence this behavior will help shed light on the factors driving its evolution. Here, we simultaneously address multiple socio-ecological conditions potentially influencing female infidelity in a natural population of the cooperatively breeding Seychelles warbler, *Acrocephalus sechellensis*. Our contained study population has been monitored for more than 25 years, enabling us to capture variation in socio-ecological conditions between individuals and across time and to accurately assign parentage. We test hypotheses predicting the influence of territory quality, breeding density and synchrony, group size and composition (number and sex of subordinates), and inbreeding avoidance on female infidelity. We find that a larger group size promotes the likelihood of extra-pair paternity in offspring from both dominant and subordinate females, but this paternity is almost always gained by dominant males from outside the group (not by subordinate males within the group). Higher relatedness between a mother and the dominant male in her group also results in more extra-pair paternity—but only for subordinate females—and this does not prevent inbreeding occurring in this population. Our findings highlight the role of social conditions favoring infidelity and contribute toward understanding the evolution of this enigmatic behavior.

## Teaser

Infidelity is common in animals that form breeding pairs. Why this behavior occurs—and varies in frequency between species, populations, and individuals—remains unresolved. We investigate the role of social, demographic, and ecological factors on the occurrence of infidelity in Seychelles warblers. In this species, dominant pairs are often joined by subordinates to form breeding groups. We find that social conditions—group size and pair relatedness—promote female infidelity, but this infidelity almost always occurs with extra-group males.

## INTRODUCTION

The occurrence of extra-pair paternity (EPP: genetic promiscuity) within socially monogamous breeding systems is widespread (birds: e.g., Richardson and Burke 1999; Foerster et al. 2003; mammals: e.g., Schulke et al. 2004; Kitchen et al. 2006; Munshi-South 2007; fish: e.g., Lee-Jenkins et al. 2015; Lee et al. 2016; Bose et al. 2018; reptiles: e.g., Bull et al. 1998; While et al. 2009; insects: e.g., Dillard 2017), but its evolution remains enigmatic, despite decades of research (Griffith et al. 2002; Forstmeier et al. 2014; Taylor et al. 2014). Levels of EPP are highly variable, not only between different individuals, populations, and species, but also across time (Petrie and Kempenaers 1998; Griffith 2000; Dietrich et al. 2004; Schroeder et al. 2016). This variation may be partly responsible for the ongoing lack of clarity surrounding the evolution of this phenomenon. Different extrinsic conditions—ecological, demographic, and social—may play a key role in this variability, with certain factors promoting, and others suppressing EPP (Griffith et al. 2002; Westneat and Stewart 2003; Isvaran and Clutton-Brock 2007; Cohas and Allainé 2009; Brouwer et al. 2017). However, across taxa, which conditions affect EPP, and how, is still not fully understood (see reviews: Griffith et al. 2002; Isvaran and Clutton-Brock 2007; Uller and Olsson 2008; Hsu et al. 2015). A potential problem is that the influence of socio-ecological factors on EPP has been investigated extensively in avian species, and to a lesser extent in mammals, while other taxa have received very little attention. This narrow taxonomic focus may have provided results which are limited by a lack of phylogenetic diversity. Importantly, up until recently, most studies investigating the factors influencing EPP have focused on just one or very few hypotheses. This may have hampered knowledge on the relative importance of different conditions shaping levels of EPP (Brouwer et al. 2017).

Various ecological, demographic, and social conditions have been proposed to influence EPP within socially monogamous systems, though the evidence for these hypotheses remains ambiguous (reviewed in Griffith et al. 2002; Westneat and Stewart 2003; Ackay and Roughgarden 2007). For example, habitat quality (i.e., resource availability) has been predicted to influence EPP in 2 opposing ways. According to the constrained female hypothesis (Gowaty 1996), in species with biparental brood provisioning, females in high-quality territories can afford to be unfaithful because high resource availability should compensate for any reduction in paternal care by males who lose (confidence in) paternity. Alternatively, if females gain extra resources by mating with more than one male (e.g., access to the extra-pair male’s territory for feeding), EPP may increase in low-quality areas (Gray 1997). Evidence for these alternative hypotheses is mixed, with some studies finding a positive (e.g., Hoi-Leitner et al. 1999; Charmantier and Blondel 2003) and others a negative (e.g., Vaclav et al. 2003; Rubenstein 2007) territory quality–EPP relationship.

Breeding density (i.e., the number of reproductively mature individuals in an area) has been predicted to increase potential mate encounter rate and, consequently, EPP frequency (Alexander 1974; Birkhead 1978; Gladstone 1979; Moller and Birkhead 1993). Research assessing the effect of breeding density on EPP has provided conflicting results, with studies showing a positive correlation (e.g., Moller 1991; Richardson and Burke 2001; Stewart et al. 2010; Annavi et al. 2014; Hellmann et al. 2015), a negative correlation (e.g., Barber et al. 1996; Verboven and Mateman 1997; Moore et al. 1999; Václav and Hoi 2002) or no relationship (e.g., Rätti et al. 2001).

Another factor hypothesized to influence EPP is breeding synchrony, that is, the overlap of female fertility within a population. The male assessment hypothesis predicts that breeding synchrony increases EPP by enabling females to compare potential mates more effectively (Stutchbury and Morton 1995). In contrast, the male trade-off hypothesis expects higher synchrony to decrease EPP because males will face a higher trade-off between mate-guarding and seeking copulations with extra-pair females (Westneat 1990). Studies addressing the relationship between breeding synchrony and EPP have provided mixed evidence so far (positive relationship: e.g., Stutchbury et al. 1997, 1998; negative relationship: e.g., Saino et al. 1999; van Dongen and Mulder 2009; no relationship: e.g., Kempenaers et al. 1997; Hoi-Leitner et al. 1999; Richardson and Burke 2001; Arlt et al. 2004; Brouwer et al. 2017).

In group-breeding taxa, characteristics of the social group have also been predicted to influence genetic promiscuity. In cooperative breeders in which groups consist of a dominant pair and non-reproducing helpers, the proportion of EPP may increase when more helpers are present. Helpers may liberate females from their dependency on their social males, that is, by mitigating the impact of those males reducing their parental care if they lose (confidence in) paternity (Mulder et al. 1994). For example, in many *Maluridae* species, EPP frequency was shown to increase with the number of helpers (Mulder et al. 1994; Webster et al. 2004; Brouwer et al. 2017; Hajduk et al. 2018; but see: Johnson and Pruett-Jones 2018). In some species, within-group EPP may occur because it leads to increased overall care to the brood and thus load-lightening for the dominant individuals, as a result of investment by those subordinates gaining paternity (Davies 1992). Evidence for this exists in several species, including dunnocks, *Prunella modularis* (Davies et al. 1996), and cichlids, *Neolamprogus pulcher* (Bruintjes et al. 2011).

In taxa in which social groups include multiple breeding males and females, genetic promiscuity can be considered in terms of extra-group paternity (EGP), resulting from the fertilization of females by males outside the social group. Group size has been predicted to increase the EGP frequency in such taxa, via a reduction in a male’s ability to monopolize females (Van Noordwijk and Van Schaik 2004). In particular, it has been predicted that when there are more females in a group, males will be less effective in controlling or defending individual females (Isvaran and Clutton-Brock 2007). On the other hand, male group size has been expected to reduce the proportion of EGP, because of increased male monopolization of females (Van Noordwijk and Van Schaik 2004). To date, the relationship between EGP and group size/composition has not been resolved (see e.g., Van Noordwijk and Van Schaik 2004; Isvaran and Clutton-Brock 2007; Rubenstein 2007; Ruiz-Lambides et al. 2017).

The relatedness of the male and female in a pair has also been predicted to influence patterns of EPP. According to the inbreeding avoidance hypothesis females should seek extra-pair fertilizations when they are closely related to their social males to increase offspring heterozygosity and fitness (Brooker et al. 1990; Blomqvist et al. 2002). Evidence for this hypothesis is mixed, with some studies showing a positive relationship between pair relatedness and EPP (e.g., Blomqvist et al. 2002; Eimes et al. 2005; Arct et al. 2015) and others finding no such relationship (e.g., Schmoll et al. 2005; Ackay and Roughgarden 2007; Edly-Wright et al. 2007; Barati et al. 2018).

Here, we simultaneously assess the relationship between multiple socio-ecological factors and female infidelity using data from a long-term study of an isolated population of Seychelles warblers, *Acrocephalus sechellensis* (see Table 1 for details). The Seychelles warbler is a socially monogamous, yet genetically promiscuous species, in which extra-pair fertilizations are common; circa 44% of offspring are sired by males other than the social male (Richardson et al. 2001; Hadfield et al. 2006). Individuals are territorial and live either in pairs or in groups consisting of a dominant pair and subordinate birds (helpers and non-helpers; Komdeur 1992; Richardson et al. 2002, 2007). Subordinate females sometimes lay eggs in the dominant females’ nest, accounting for circa 15% of offspring in the population (Richardson et al. 2001; Hadfield et al. 2006). Almost all paternity is gained by dominant males, with just 2% of offspring being sired by subordinate males within the group (Richardson et al. 2001; Hadfield et al. 2006), usually those transitioning toward dominant status (Dugdale HL, unpublished data), while there are no recorded cases of EGP gained by subordinates (Richardson et al. 2001). Hence, EPP in this species is almost completely EGP, that is, the result of fertilizations by males outside the group.

**Table 1:** List of socio-ecological parameters (1–9) and an additional control factor (10), how these factors are estimated, and the predictions about how they may influence EGP in the Seychelles warbler

Parameter	Estimation	Predicted effect on EGP
1. Territory quality	Invertebrate prey availability per territory (based on arthropod counts, vegetation cover, and territory size)	Increase in EGP if resource abundance compensates for male retaliation (i.e., care reduction)
2. Local breeding density (males)	Number of neighboring dominant males (i.e., in territories adjacent to the focal territory)^a^	Increase in EGP via higher mate encounter rate
3. Population breeding density (males)	Number of dominant males on Cousin	Increase in EGP via higher mate encounter rate
4. Local breeding synchrony	Number of neighboring dominant females whose fertile period (6–0 days preceding egg laying; Eikenaar 2006) overlaps that of the focal female	Decrease in EGP due to male trade-off between mate-guarding and pursuit of EGP (a trade-off is present in Seychelles warblers; Eikenaar 2006)
5. Population breeding synchrony	Number of dominant females in the population whose fertile period overlaps that of the focal female	Reduction in EGP due to male trade-off between mate-guarding and EGP pursuit
6. Group size	Number of independent birds (≥3 months old) in the focal territory	Increase in EGP due to a reduction in mate-guarding (via a “confusion effect”)
7. Reproductively mature subordinates	All: Number of subordinates (helpers and nonhelpers) ≥8 months old (other than the mother) in the focal territory	Increase in EGP due to a reduction in mate-guarding effectiveness (via different mechanisms for mature males vs. females, see below).
	Males: Presence of male subordinates ≥8 months old	Males: increase in EGP due to a trade-off between subordinate male suppression and mate-guarding (dominant males physiologically suppress subordinate males; Brouwer et al. 2009a)
	Females: Presence of female subordinates ≥8 months old (other than the mother)	Females: increase in EGP via difficulty in controlling individual females when >1 are present
8. Helpers	Number of helpers in the focal territory (other than the mother)	Increase in EGP if helpers compensate for male retaliation (helpers provide load-lightning in Seychelles warblers; van Boheemen et al. 2019)
9. Pairwise genetic relatedness (*R*)	Mother-social (dominant) male genetic relatedness using the Queller and Goodnight (1989) estimation	Increase in EGP via inbreeding avoidance
10. Clutch size (per female)	Presence/absence of >1 offspring produced by the same female in the same nest	Increase in EGP via higher chance of at least one offspring being extra-group

See Supplementary Table S1 for details on the distribution of each socio-ecological variable.

^a^Territories are inhabited by a dominant male and a dominant female and, in 30–50% of cases, also by subordinate individuals of either sex. Extra-group offspring are almost always sired by dominant males, which are often from adjacent territories (Richardson et al. 2001; Hadfield et al. 2006).

Our study population of the Seychelles warbler is confined to a single small island (Cousin, Seychelles) and displays virtually no inter-island dispersal (Komdeur et al. 2004, 2017). Since 1997, >96% of Seychelles warblers on this island have been individually color-ringed and blood-sampled for sexing and parentage assignment (Brouwer et al. 2010). These features of our study population enable accurate parentage, reproductive output and survival estimates, unconfounded by migration in or out of the population. The long-term nature of the monitoring also enables us to capture changes in socio-ecological conditions across the lifetime of individual birds. The simultaneous assessment of multiple socio-ecological conditions in this study system therefore provides a powerful approach to reveal the factors influencing EGP.

## METHODS

### Study system

The Seychelles warbler is an insectivorous passerine endemic to the Seychelles archipelago. The population on Cousin Island (29 ha, 04°20′S, 55°40′E) has been monitored since 1981 (Komdeur 1992; Richardson et al. 2002; Wright et al. 2014; Bebbington et al. 2017). Monitoring efforts were intensified since 1997: virtually all breeding attempts have been followed every year during the major breeding season (June–September) and, often, during the minor breeding season (January–March, Richardson et al. 2002, 2010). Every year, as many individuals as possible were caught with mist-nets, blood sampled (ca. 25 μL) and, if caught for the first time, given a unique ring combination (a British Trust for Ornithology metal ring and 3 color rings). As inter-island dispersal is virtually absent (<0.1%; Komdeur et al. 2004, 2017) and resighting probability is very high (ca. 92% for individuals up to 2 years old and 98% for older birds), individuals that were not observed more than 2 consecutive seasons could be confidently assumed to be dead (Brouwer et al. 2006, 2010).

Blood samples were used for molecular sexing, following Griffiths et al. (1998), and genotyping using 30 microsatellites (Richardson et al. 2001; Spurgin et al. 2014). Parentage assignment was completed using MasterBayes 2.52 (for details, see Edwards et al. 2018). Pairwise genetic relatedness between each mother (dominant or subordinate) and the dominant male in her group was calculated based on the microsatellite data by implementing Queller and Goodnight’s (1989) estimation of relatedness with the R package “related” v. 0.8 (Pew et al. 2015).

Seychelles warblers are territorial: individuals normally pair up, reside in and defend the same territory for life (Komdeur 1992; Richardson et al. 2007). In about 30% (1997–1999) or 50% (2003–2014) of territories, the dominant pair is joined by one or more subordinates of either sex (Komdeur 1992; Richardson et al. 2002, 2007; Kingma et al. 2016). Subordinates are often, but not always, offspring that delay dispersal from their natal territory (Kingma et al. 2016). Throughout each breeding season, censuses were performed in all territories to assign group membership and determine individual status. Groups were identified based on foraging location, proximity, and non-aggressive interactions between individuals. Within groups, dominant breeders were identified via clear courtship and pair behavior and subordinates were assigned helper or non-helper status, based on whether they contributed to raising young in the territory (Komdeur 1992; Richardson et al. 2002).

Seychelles warblers feed on arthropods, 98% of which are taken from the underside of leaves (Komdeur 1991). Hence, territory quality was calculated in terms of arthropod availability, estimated using a combination of arthropod counts, vegetation cover, and territory size (Brouwer et al. 2009b). Reproduction is seasonally limited by arthropod availability and is energetically expensive, as both sexes feed young for circa 3 (and sometimes up to 4) months after hatching (Komdeur 1996; Komdeur et al. 2017).

### Dataset and parameter estimation

We assessed the relationship between 9 different socio-ecological parameters and the probability that young are sired by extra-group males (EGP likelihood). We obtained parentage data from previous work (Richardson et al. 2001; Hadfield et al. 2006; Spurgin et al. 2014; Edwards et al. 2017) for individuals born on Cousin during major breeding seasons between 1997 and 2014. A dataset consisting of offspring and the socio-ecological factors associated with each offspring’s natal group during the individual’s hatching season was compiled (summarized in Table 1). We excluded offspring sired by within-group subordinate males (i.e., cases of within-group EPP) and young produced by extra-group subordinate males, as these were both very rare (9 and 16 out of 990 offspring, respectively).

### Statistical analyses

We separately assessed the effect of socio-ecological parameters on EGP likelihood of offspring from dominant (*n* = 861) and subordinate (*n* = 104) females, as these may differ in terms of the most influential factors and their interactions. For simplicity, we refer to the EGP of offspring from dominant or subordinate females as “dominant female EGP” or “subordinate female EGP,” respectively (EGP of offspring is the result of female infidelity). Information on all parameters was not available for all offspring, so we subdivided the dominant female data set into 3 subsets with no missing values. Subset A (*n* = 816) was created by including all socio-ecological factors except breeding synchrony and clutch size, as these could be estimated only for a smaller number (see below) of offspring with the relevant nest information available. Territory quality data was unavailable for <25% of offspring (due to shorter fieldwork periods in a couple of years), but was included in subset A, with missing data points extrapolated from adjacent seasons (mean territory quality value of the previous and the following major breeding season, following Brouwer et al. 2006). To test that this extrapolation did not affect results, we compiled a second subset (B, *n* = 636), consisting of cases with complete territory quality (non-extrapolated) data and all other data, except breeding synchrony and clutch size. We then created a third subset (C, *n* = 356) with all available nest information, to address the effect of breeding synchrony and to control for a potential effect of clutch size. We did not subset the subordinate female dataset due to sample size limitations.

We analyzed each subset/dataset with an information-theoretic approach (model averaging) using R (v.3.4.0), based on the construction of global generalized mixed effect models (GLMMs) containing all noncollinear (VIF ≤ 3) variables of interest as fixed effects (package *lme4* 1.1–12; Bates et al. 2015). To assess the effect of group size (which included immature birds) and of just the number of reproductively mature subordinates (which were correlated), we built 2 sets of models, each including one of these predictors with all other fixed effects, and ran separate analyses. It was possible to model the number of helpers alongside group size or the number of mature subordinates because the number of helpers was not collinear with either of the latter 2 variables (VIF ≤ 3). Even though the number of mature subordinates included helpers and non-helpers, we modeled the number of helpers alongside that of all mature subordinates, rather than with the number of non-helping subordinates. We did this because we had specific predictions on the effect that helpers and mature subordinates may have on EGP (Table 1), while we had no predictions for non-helping subordinates. Global GLMMs were built with a binomial error structure, standardization (scaling and centering) of continuous predictors, and the “*Bobyqa*” nonlinear optimization (Powell 2009) for model convergence. To eliminate pseudo-replication, we included the following random effects: year, mother identity, and social male identity. In analyses of the subordinate dataset featuring group size/helpers/mature subordinates split by sex, we combined mother identity and social male identity in one random effect (social pair identity), to avoid model overfitting. We used this combined random effect also when analyzing subset C, to aid model convergence. Here, we also included nest identity, since nest information was available, and found that this random effect explained zero variance (see Results section). From each global model, we built competing models based on all possible fixed effect combinations, ranked these models by AIC_c_ scores and assigned them Akaike weights (ω_m_) based on such scores (package *MuMIn* 1.40.0, Barton 2017). All models with AIC_c_ within 2 of the best model AIC_c_ (ΔAIC_c_ ≤ 2) were included in the top model set. We calculated full averaged estimates for each variable, that is, model-weighted averages of predictor estimates over all top set models, including models that did not contain the predictor (in such models the estimate was zero). We also calculated the relative importance (ω_p_) of explanatory variables, that is, the sum of Akaike weights of all top set models containing the variable. Since models where ΔAIC_c_ ranges 2–7 may have some support (Burnham et al. 2011), we reanalyzed our data using a top model set cutoff of 7 ΔAIC_c_ and found results to be consistent. As the subordinate mother dataset was smaller—101 offspring with no missing data (ignoring nest information)—and nest-related data were available only for 49 offspring, we analyzed all variables of interest, except breeding synchrony and clutch size, in relation to subordinate female EGP likelihood (Table 3).

## RESULTS

We obtained parentage data for 990 offspring: 884 produced by dominant females and 106 by subordinate females. Out of all 990 offspring, 965 were sired by dominant males and 25 by subordinate males. Since cases of within-group and extra-group subordinate paternity were both very rare (9 and 16 offspring, respectively), we excluded these from our analyses of EGP. The overall frequency of EGP was 41% (395/965). There was a tendency for subordinate mothers to have a higher proportion of offspring with EGP, 51% (53/104), than dominant mothers, 40% (341/861), but this did not reach statistical significance (GLMM: *β*_Mother status_ = 0.46 ± 0.26, *P* = 0.07; Supplementary Table S2). Dominant females produced 89% of all offspring and subordinate females 11%. However, only 32% of territories included ≥1 reproductively mature (i.e., ≥8 months old) female subordinate. In these territories, 66% of all offspring had a dominant mother and 34% a subordinate mother. The genetic relatedness (*R*) between a female and the dominant male in her territory did not differ with respect to female status (LM: *β*_Mother status_ = 0.02 ± 0.03, *P* = 0.64).

### Dominant female EGP

**Figure 1:**
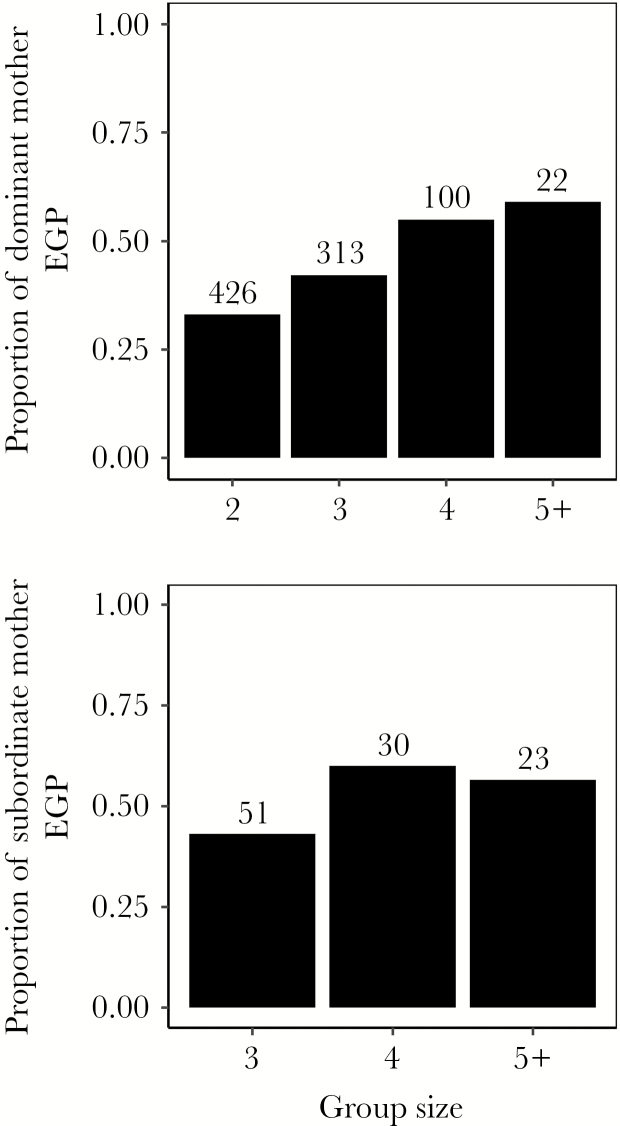
The proportion of EGP of offspring with dominant (top graph) and subordinate (bottom graph) mothers in relation to group size in the Seychelles warbler. The proportion of extra-group offspring produced by dominant (top graph) and subordinate (bottom graph) females is higher in larger groups. Clutch size is usually one (but ca. 33% of nests have 2 or 3 eggs, usually as a result of cobreeding) and most females produce one offspring per nest.

Dominant female EGP increased in larger groups (Figure 1, Table 2) and both male and female group size had similar (positive) effects (Supplementary Table S3). Dominant female EGP was also higher in territories with more mature subordinates (Supplementary Table S4), though group size was a better predictor of EGP than the number of mature subordinates (the AIC_c_ score of the best overall model containing group size was 6 units lower than the AIC_c_ of the best overall model including the number of mature subordinates, Supplementary Tables S13 and S15). Male and female mature subordinates both had positive effects on dominant female EGP (Supplementary Table S5); the analysis including these as 2 separate predictors gave a best overall model with a slightly weaker AIC_c_ than the best overall model from the analysis of all subordinates combined (Supplementary Tables S15 and S16).

**Table 2:** Model-averaged parameters: the effect of socio-ecological predictors—including group size—on the likelihood of EGP in offspring from dominant females in the Seychelles warbler (subset A)

Fixed term	β	95% CI	ω_p_
(Intercept)	−0.47	−0.66, −0.27	—
***Group size***	***0.35***	***0.17, 0.53***	***1.00***
Population breeding density	−0.07	−0.24, 0.11	0.53
Pairwise relatedness	0.06	−0.12, 0.24	0.46
Territory quality	0.01	−0.09, 0.11	0.25
Number of helpers	−0.01	−0.11, 0.09	0.19
Local breeding density	—	—	—
**Random term**	**σ** ^**2**^	**95% CI**	**N**
Mother ID	0.15	0.00, 0.86	313
***Social male ID***	***0.58***	***0.31, 1.10***	***311***
Year	0.00	0.00, 0.25	17

Response: Dominant female EGP likelihood (*n* = 816 offspring). Candidate models: 64. Top set models: 11 (see Supplementary Table S13 for details). Full model-averaged estimates (β), 95% confidence intervals (CIs), and relative importance (ω_p_) are shown for all socio-ecological predictors featuring in the top model set (ΔAIC_c_ ≤ 2). Random effect variances (σ^2^) and their 95% CIs in the best model are also shown. Predictors whose CIs do not overlap with zero are given in bold italics.

**Table 3:** Model-averaged parameters: the effect of socio-ecological predictors—including group size—on the likelihood of EGP in offspring from subordinate mothers in the Seychelles warbler

Fixed term	β	95% CI	ω_p_
(Intercept)	0.10	−0.52, 0.73	—
Group size	0.71	−0.04, 1.46	1.00
***Pairwise relatedness***	***0.71***	***0.05, 1.36***	***1.00***
Number of helpers	−0.10	−0.57, 0.37	0.28
Territory quality	0.05	−0.34, 0.45	0.21
Population breeding density	—	—	—
Local breeding density	—	—	—
**Random term**	**σ** ^**2**^	**95% CI**	**N**
Mother ID	1.59	0.00, 2.21	53
Social male ID	0.00	0.00, 2.91	58
Year	0.00	0.00, 0.97	16

Response: subordinate female EGP likelihood (*n* = 101 offspring). Candidate models: 64. Top set models: 3 (see Supplementary Table S20 for details). Full model-averaged estimates (β), 95% confidence intervals (CIs), and relative importance (ω_p_) are shown for all socio-ecological predictors featuring in the top model set (ΔAIC_c_ ≤ 2). Random effect variances (σ^2^) and their 95% CIs in the best model are also shown. Predictors whose CIs do not overlap with zero are given in bold italics.

Dominant female EGP was not related to the number of helpers (or whether male and female helpers were present) or any of the other variables tested in subset A (population breeding density, local breeding density, territory quality and *R*; Table 2, Supplementary Table 6). The territory quality extrapolation did not affect results (see subset B analysis, Supplementary Table S7), which were consistent across subsets with or without the extrapolated data. Population and local breeding synchrony, their interaction with population and local breeding density, respectively, and clutch size also did not influence the likelihood of dominant female EGP (see subset C analysis, Supplementary Table S8). Social male and social pair identity were the only random effects to explain variation in dominant female EGP with high confidence (i.e., with 95% CIs not overlapping zero, Table 2, Supplementary Tables S2–S6 and S8) and explained circa 12–14% and 20%, respectively, of the total variance in dominant female EGP.

### Subordinate female EGP

**Figure 2:**
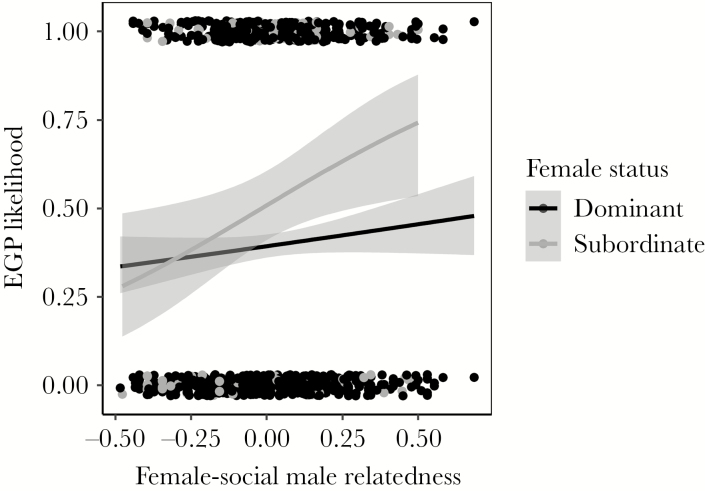
EGP likelihood in relation to pairwise relatedness (*R*) between each mother (dominant or subordinate) and the dominant male in the territory (social male) in the Seychelles warbler. Likelihood of offspring being sired by extra-group males for dominant mothers (in black, *n* = 861) and subordinate mothers (in gray, *n* = 104) in relation to the genetic relatedness between the mother and the social male. The positive relationship is significant for subordinate mothers but not for dominant mothers (Tables 2 and 3).

Subordinate female EGP was positively associated to both relatedness (*R*) and group size (Table 3, Figures 1 and 2). Only *R* was conventionally significant (the 95% CI of *R* did not overlap zero), but both group size and *R* had a ω_p_ of 1.00 (and the 90% CI of group size did not overlap zero). These results suggest that group size also influenced subordinate female EGP, but that power was limited in our much smaller sample of offspring from subordinate females. All other variables tested, including male and female group size, the number of mature subordinates and helpers (or whether male and female subordinates and helpers were present, respectively), had ω_p_ < 0.90 and CIs overlapping zero (Supplementary Tables S9–S12). When testing for the effect of the number of mature subordinates (or whether male and female subordinates were present), the 95% CI of *R* overlapped zero and its ω_p_ dropped below 1.00, possibly due to lack of power in the small sample. However, *R* was still a highly important factor in the models (Supplementary Tables S10 and S11). Overall, our results suggest that the likelihood of subordinate female EGP is related to *R*. Using the same microsatellite markers for the estimation of relatedness and the assignment of parentage could result in inadvertent bias, leading to the detection of a false positive association between relatedness and extra-pair paternity (Wetzel and Westneat 2009). However, we only found a positive *R-*EGP relationship in the small subset containing offspring of subordinate females, and not in the large subset with offspring of dominant females, even though the latter subset had much more power. Also, we know that the positive association between *R* and EGP in the subordinate subset was not caused by overall higher levels of female–male relatedness (*R* did not differ in relation to female status). Therefore, it is highly unlikely that inadvertent bias influenced these results. All random effects tested had 95% CIs overlapping zero (Table 3, Supplementary Tables S9–S12).

## DISCUSSION

In Seychelles warblers, 41% of offspring resulted from extra-group fertilizations, of which 96% were sired by dominant males. Here, we focused on analyzing the relationship between multiple social, demographic, and ecological factors and female EGP. The proportion of EGP in offspring from dominant (40%) and subordinate (51%) females tended to differ, but this difference was not statistically significant. Both dominant and subordinate female EGP increased with group size. Importantly, the numbers of either male or female group members in a territory had similar positive effects on EGP. Furthermore, overall group size (including reproductively immature birds), was a better predictor of EGP than the number of mature subordinates in a territory. Although the relatedness of dominant and subordinate females to the dominant male did not differ significantly, female-dominant male relatedness was only a positive predictor of EGP likelihood for subordinate mothers. None of the other factors tested (i.e., breeding density, breeding synchrony, number of helpers, territory quality, or clutch size) were found to influence EGP.

### Group features and EGP

In group-breeding species, the number of adults within the group has been predicted to impair a male’s ability to monopolize individual females and, consequently, to increase the proportion of EGP (Van Noordwijk and Van Schaik 2004). Past research addressing this hypothesis has often failed to provide clear supporting evidence (see e.g., Van Noordwijk and Van Schaik 2004; Rubenstein 2007; Ruiz-Lambides et al. 2017). The same applies to studies specifically testing for an effect of the number of adult males in the group (e.g., Durrant and Hughes 2005; Isvaran and Clutton-Brock 2007; but see: Annavi et al. 2014), which may reduce EGP via improved control or defense of females (Van Noordwijk and Van Schaik 2004). Evidence that the number of adult females in the group leads to higher rates of EGP (because it impairs male monopolization of individual females; Van Noordwijk and Van Schaik 2004) has perhaps found more support, though this was not always the case. For instance, a meta-analysis of group-living mammal species found a positive correlation between EGP frequency and the number of adult females per group (Isvaran and Clutton-Brock 2007), and a recent study on rhesus macaques, *Macaca mulatta*, found that EGP increased with the number of adult females, but only in large groups (Ruiz-Lambides et al. 2017). In contrast, work on European badgers, *Meles meles*, provided the opposite result (i.e., a negative relationship between adult female group size and EGP; Annavi et al. 2014). Studies on polygynous species have also provided mixed results relating to the effect of harem size on the proportion of extra-harem paternity (e.g., Cordero et al. 2003 versus Sousa and Westneat 2013).

As predicted (Table 1), the number (or presence) of mature subordinates (of either sex) in a territory, had a similar positive effect on dominant female EGP. However, total group size, which also includes reproductively immature independent birds (old fledglings and subadults), better predicted dominant and subordinate female EGP than only the number of sexually mature subordinates. This, and the fact that the number of either male or female subordinates influenced EGP similarly, indicates that female EGP is increased by group size, rather than the group’s composition acting via different mechanisms through male and female subordinates. One explanation for the group size–EGP relationship may be that, in territories with more residents, dominant males may be less effective at keeping track of, and closely mate-guarding, the fertile female(s). In the future it might be possible to test if group size influences mate-guarding rate.

Another plausible explanation for the positive effect of group size on EGP is that group size reflects the former reproductive success of the breeding female(s) and is used as a social cue by males seeking EGP. Studies have shown that reproductive success can be adopted by conspecifics as public information for mate and habitat selection (e.g., Drullion and Dubois 2011; Pärt et al. 2011). If Seychelles warblers used group size as public information indicating breeder and/or territory productivity, males would preferentially seek extra-pair fertilizations with females living in larger groups, thus leading to higher EGP in larger groups. In Seychelles warblers, males were observed intruding into territories to seek extra-group copulations, but whether the probability of this happening is linked to group size is unknown and needs investigating.

The effect of group size may be linked to the age and/or quality of female breeders, and a corresponding increase in the amount of young these females produce. Since offspring often delay dispersal from the natal group (Kingma et al. 2016), older and/or higher quality female breeders will more likely reside in larger groups (with a higher number of retained offspring). It is therefore possible that older (more experienced) and/or higher-quality females, which live in larger groups, are better at evading mate-guarding and at obtaining extra-group fertilizations. Also, EGP is likely modulated by social-male characteristics, as supported by the fact that social male and social pair identity explained 12–14% and 20% of the variation in EGP likelihood, respectively, at least for dominant females. This suggests that females paired with certain males are more unfaithful than others. Numerous studies have attempted to find individual traits related to the loss or gain of extra-pair paternity by males, but the evidence remains unclear (see reviews: Griffith et al. 2002; Ackay and Roughgarden 2007). A previous study in the Seychelles warbler showed that social males with low MHC diversity lost more paternity than those with higher MHC diversity (Richardson et al. 2005). Moreover, in a translocated Seychelles warbler population, where female choice was not constrained by territory availability, older and more heterozygous males were more likely to be paired (Wright et al. 2015). We also know that dominant Seychelles warbler males, which are on average older than subordinate males, gain most within-group and extra-group paternity (Richardson et al. 2001; Hadfield et al. 2006). Across many species, it is generally true that older males obtain most of the paternity (Richardson and Burke 1999; Ackay and Roughgarden 2007; Hsu et al. 2015). To understand this further in Seychelles warblers, it would be helpful to assess any potential relationship between EGP and maternal or paternal traits, including age and features associated with individual fitness, as well as with the pairwise combination of such traits.

Several studies on cooperative species have shown that helpers provide load-lightening for dominants, that is, allowing them to reduce their work rate and investment into young (e.g., MacColl and Hatchwell 2003; Clutton-Brock et al. 2004; Russell et al. 2008; Bruintjes et al. 2013; Zöttl et al. 2013). Having helpers who provide parental care may liberate females from the constraints imposed on them by reduced parental care from pair males who lose (certainty of) paternity (Mulder et al. 1994). Evidence supporting this prediction comes from studies of fairy wren species, showing that EPP increased with the number of helpers in the group (Mulder et al. 1994; Webster et al. 2004; Brouwer et al. 2017; Hajduk et al. 2018; but see: Johnson and Pruett-Jones 2018). In Seychelles warblers, helpers facilitate the load-lightening of dominant females (van Boheemen et al. 2019) and increase offspring survival (Brouwer et al. 2012). However, we found that the number (or presence) of helpers (of either sex) had no effect on EGP in either dominant or subordinate females. A possible explanation for this null result is the absence of male retaliation in Seychelles warblers. In this scenario, females are not constrained by social males and therefore do not need to be liberated by helpers. Comparisons of parental care (e.g., feeding rates to nestlings) undertaken by cuckolded and non-cuckolded males would be necessary to confirm this.

### Inbreeding avoidance via EGP

Our results provide limited support for the idea that EGP may be part of an inbreeding avoidance mechanism, that is, that females who are closely related to their social male avoid inbreeding by mating with extra-group males. While some studies have found a positive effect of pair relatedness on EPP (e.g., Blomqvist et al. 2002; Cohas et al. 2006; Freeman-Gallant et al. 2006; Leclaire et al. 2013), others have shown no effect (e.g., Schmoll et al. 2005; Edly-Wright et al. 2007; Barati et al. 2018). Mixed evidence has resulted also from meta-analyses (in favor: Arct et al. 2015; against: Ackay and Roughgarden 2007) and from research on polygynandrous species, which addressed the effect of female–male relatedness (within a group) on EGP levels (e.g., Nichols et al. 2015 versus Ruiz-Lambides et al. 2018). In the Seychelles warbler, we only detected a positive relationship between genetic relatedness and EGP likelihood for subordinate mothers. This result concurs with a previous, smaller study in Seychelles warblers that did not find an effect of relatedness on EGP across all females, but did show that extra-group young of subordinate females were less inbred than their within-group offspring (Richardson et al. 2004). This study also found that inbreeding had a negative inter-generational impact on offspring survival via maternal effects (Richardson et al. 2004), a result confirmed using telomeres as biomarkers in a much larger recent study (Bebbington et al. 2016). A possible explanation as to why only subordinate, but not dominant, females may avoid inbreeding via EGP, is that dominant females are the primary focus of mate-guarding (Komdeur et al. 1999). Hence, subordinates may have more freedom to pursue extra-group fertilizations, which they may be more likely to seek when they are highly related to the dominant male in the territory. However, given that close inbreeding does occur in Seychelles warblers (Richardson et al. 2004) and 40% of offspring from dominant females have EGP, there must be other reasons why dominant females do not avoid inbreeding.

Richardson et al. (2004) showed that subordinate mothers were more related to the dominant male than were dominant mothers and that the proportion of EGP for subordinate females was higher than for dominant females. However, we detected no difference in female–social male relatedness in the present study. This is possibly due to the high frequency of EGP in Seychelles warblers. Even if subordinate females are offspring that have remained in their natal territory, which is not always the case (Kingma et al. 2016; Groenewoud et al. 2018), they have a 41% chance of being sired by an extra-group male. Moreover, mortality and the replacement of dominant individuals does occur over time, thus further decreasing the chance of dominant males being the fathers of co-breeding subordinates (see Kingma et al., in preparation). Also, dominant females can be highly related to the social male if they have inherited dominance in their natal territory and have ended up being paired with their own father (Eikenaar et al. 2008).

### Territory quality, demographic factors, and EGP

Territory quality has been predicted to influence EPP positively—high territory quality may promote infidelity by compensating for costs (reduced paternal care) imposed by male retaliation against unfaithful females (Gowaty 1996)—or negatively—low territory quality may increase EPP if females can gain extra resources from extra-pair males (Gray 1997). Low territory quality may also result in females moving further afield while seeking resources, therefore increasing encounters with extra-pair males and, consequently, EPP levels. The relationship between territory quality and EPP, however, is far from being resolved, with studies showing either a positive (e.g., Hoi-Leitner et al. 1999; Charmantier and Blondel 2003) or a negative relationship (e.g., Vaclav et al. 2003; Rubenstein 2007). In our study, territory quality does not seem to influence EGP likelihood. It is possible that male Seychelles warblers do not retaliate (i.e., reduce parental care) when they lose (confidence in) paternity and that infidelity does not cause females significant energetic costs, which would be compensated for by high habitat quality.

Breeding density has been predicted to promote EPP by increasing mate encounter rate (Alexander 1974; Birkhead 1978; Gladstone 1979; Moller and Birkhead 1993). Comparisons across species have provided little evidence for any such correlation (Westneat and Sherman 1997; Griffith et al. 2002). However, the relationship seems to hold in various correlative studies focusing on individual species (e.g., Moller 1991; Richardson and Burke 2001; Soucy and Travis 2003; Mayer and Pasinelli 2013; Annavi et al. 2014; but see e.g., Barber et al. 1996; Verboven and Mateman 1997; Tarof and Stutchbury 1998; Moore et al. 1999). The few studies which have experimentally manipulated breeding density also provided mixed evidence, finding either a positive breeding density–EPP correlation (Gowaty and Bridges 1991; Charmantier and Perret 2004; Stewart et al. 2010), no relationship (Rätti et al. 2001) or a negative correlation (Dunn et al. 1994; Václav and Hoi 2002). Male Seychelles warblers have been shown to adjust their reproductive physiology (van de Crommenacker et al. 2004) and mate-guarding behavior in relation to local conspecific density (Komdeur 2001; Komdeur et al. 2007), which suggests that breeding density may affect EGP. However, our study does not show a relationship between EGP likelihood and neither local nor population-wide breeding density. As population density on Cousin has been relatively stable since carrying capacity was reached in 1982 (Brouwer et al. 2009b; Komdeur et al. 2017), it may be that population breeding density is not variable enough to generate any observable effect on EGP in our study (Supplementary Table S1). Local breeding density, on the other hand, does display considerably more variation (Supplementary Table S1). In fact, even though territory boundaries are relatively stable in time, new territories can form and old ones disappear/merge with others across years, and our long data period comprises enough years (17) to capture any such changes. Considerable variation in local breeding density is present also within years, due to the location of different territories on the island. For example, central territories have many more adjacent territories compared with those bordering the coast, or next to the rocky uninhabited areas. Also, territories in invertebrate-rich areas (where territory density is higher) have more adjacent territories than those in low-quality areas. Despite this variation, local breeding density did not influence EGP. It is possible that reasons other than local (and population) breeding density drive EGP in Seychelles warblers. Alternatively, local breeding density may not be a very good predictor of EGP likelihood, as individuals may move across several territories to obtain EGP. A previous study showed that although circa 59% of extra-group fertilizations occurred with males from within 2 territories away from a female’s territory, the rest was shown to occur with males up to 6 territories away (see Richardson et al. 2001).

Breeding synchrony has been suggested as a factor either increasing EPP—by enabling females to compare potential mates more effectively (Stutchbury and Morton 1995)—or reducing EPP—by increasing the trade-off males face between mate-guarding and seeking extra-pair copulations (Westneat 1990). However, while some have found a positive (Stutchbury et al. 1997, 1998) or a negative correlation (Saino et al. 1999; van Dongen and Mulder 2009) between breeding synchrony and EPP, most studies have failed to find any relationship (e.g., Kempenaers et al. 1997; Hoi-Leitner et al. 1999; Richardson and Burke 2001; Arlt et al. 2004; Brouwer et al. 2017). Seychelles warbler males closely mate-guard their social female during her fertile period to prevent cuckoldry (Komdeur et al. 2007) and face a trade-off between mate-guarding and the pursuit of extra-pair fertilizations (Eikenaar 2006). In this species, EGP should, therefore, decrease with breeding synchrony. However, an earlier study found no such relationship (Eikenaar 2006). This was suggested to be the case because there were always plenty of non-guarding extra-group males available, due to the low local breeding synchrony and high local breeding density during the 3 years of that study (Eikenaar 2006). Despite our improved sample size (spanning 17 years), and more variation in breeding synchrony (Supplementary Table S1), we detected no effect of this demographic factor on EGP neither at the local nor at the population level.

## CONCLUSIONS

Our study investigated the effect of multiple socio-ecological conditions on EGP likelihood in a wild population. Our finding that group size was positively correlated with EGP for both dominant and subordinate females suggests that larger groups may enable females to be less faithful, though why that is remains unclear. We also found some support for the idea that infidelity functions to reduce inbreeding (inbreeding avoidance hypothesis) but only for subordinate females, who may have more opportunity to obtain EGP than dominant females. The other social, demographic, and ecological parameters tested (the number of helpers in a group, local and population breeding density, local and population breeding synchrony, territory quality) did not appear to affect EGP in the Seychelles warbler. Our study suggests that, at least in this system, other factors, possibly linked to individual traits and/or quality, may be the major determinants of EGP.

## FUNDING

This work was supported by NERC grant (NE/B504106/1 to T.A.B. and D.S.R.; H.L.D. was postdoc on this), NWO Rubicon (825.09.013) and NERC (NE/I021748/1) fellowships to H.L.D., Lucie Burgers Foundation and KNAW Schure Beijerinck Poppings grant (SBP2013/04 to H.L.D., NWO visitors grant (040.11.232 to J.K. and H.L.D.), NERC grant (NE/P011284/1 to H.L.D. and D.S.R.), NWO grants (854.11.003 and 823.01.014 to J.K.) and NERC grants (NE/F02083X/1 and NE/K005502/1 to D.S.R.).

Data Accessibility: Analyses reported in this article can be reproduced using the data provided by Raj Pant et al. (2019). The data used in this study has been uploaded in DRYAD (doi: 10.5061/dryad.h48d445).

## Supplementary Material

arz072_Suppl_Supplementary_Tables
