# Supplementary material for: Socio-ecological conditions and female infidelity in the Seychelles warbler
Source: Behav Ecol. 2019 May 30;30(5):1254–64. doi: 10.1093/beheco/arz072 (PMC6765383; doi:10.1093/beheco/arz072)
Supplement: arz072_Suppl_Supplementary_Tables [file arz072_suppl_supplementary_tables.docx]

Socio-ecological conditions and female infidelity in the Seychelles warbler

**-Supporting Information-**

**Table S1. Summary statistics of socio-ecological factors addressed in the Seychelles warbler.**

| **Variable** | **Mean** | **Median** | **Mode** | **CV** |
| --- | --- | --- | --- | --- |
| Territory quality | 41300.49 | 20387.65 | 36749.78 | 1.68 |
| Local breeding density | 4.80 | 5 | 4 | 0.32 |
| Population breeding density | 109.65 | 111 | 111 | 0.07 |
| Local breeding synchrony | 1.22 | 1 | 0 | 0.96 |
| Population breeding synchrony | 25.19 | 24 | 24 | 0.55 |
| Group size | 2.80 | 3 | 2 | 0.31 |
| Number of mature subordinates | 0.57 | 0 | 0 | 1.31 |
| Number of helpers | 0.33 | 0 | 0 | 1.70 |
| Dominant female-social male *R* | 0.03 | 0.01 | -0.01 | 6.83 |
| Subordinate female-social male *R* | 0.03 | 0.03 | 0.4 | 7.71 |

CV indicates the coefficient of variation and *R* indicates the pairwise genetic relatedness of female-social male (i.e. dominant male in the female’s group) dyads*.*

**Table S2. Output of the generalized linear mixed model (GLMM) regressing extra-group paternity (EGP) likelihood over mother status (dominant *vs* subordinate; n = 965 offspring) in the Seychelles warbler.**

| **Response** | **Fixed term** | **β** | **SE** | **p** |
| --- | --- | --- | --- | --- |
| EGP Likelihood | (Intercept) | -0.47 | 0.10 | 0.000002 |
|  | Mother status (subordinate) | 0.46 | 0.26 | 0.07 |
|  | **Random term** | **σ²** | **95% CI** | **n** |
|  | Mother ID | 0.15 | 0.00, 83 | 347 |
|  | Social male ID | 0.64 | 0.42, 1.11 | 335 |
|  | Year | 0.00 | 0.00, 0.19 | 18 |

Estimates (β), standard errors (SE) and p values (p) are shown for the intercept and the fixed effect (mother status); variance (σ²), 95% confidence intervals (CIs) and number of observations (n) are shown for each random effect.

**Table S3. Model-averaged parameters: the effect of socio-ecological predictors – including female and male group size – on the likelihood of extra-group paternity (EGP) in offspring from dominant females in the Seychelles warbler (subset A).**

| **Fixed term** | **β** | **95% CI** | **ω_p_** |
| --- | --- | --- | --- |
| (Intercept) | -0.47 | -0.67, -0.27 | - |
| **Female group size** | **0.27** | **0.09, 0.45** | **1.00** |
| **Male group size** | **0.26** | **0.09, 0.43** | **1.00** |
| Population breeding density | -0.11 | -0.30, 0.08 | 0.74 |
| Pairwise relatedness | 0.07 | -0.12, 0.26 | 0.51 |
| Territory quality | 0.009 | -0.12, 0.09 | 0.18 |
| Number of helpers | -0.01 | -0.13, 0.10 | 0.17 |
| Local breeding density | - | - | - |
| **Random term** | **σ²** | **95% CI** | **n** |
| Mother ID | 0.24 | 0.00, 0.93 | 313 |
| **Social male ID** | **0.49** | **0.16, 1.06** | **311** |
| Year | 0.00 | 0.00, 0.24 | 17 |
| **Response: dominant female EGP likelihood (n = 816 offspring).** Candidate models: 128. Top set models: 8 (see Table S14 for details). | | | |

Full model-averaged estimates (β), 95% confidence intervals (CIs) and relative importance (ω_p_) are shown for all socio-ecological predictors featuring in the top model set (ΔAICc ≤ 2). Random effect variances (σ²) and their 95% CIs in the best model are also shown. Predictors whose CIs do not overlap with zero are given in bold.

**Table S4. Model-averaged parameters: the effect of socio-ecological predictors – including the number of mature subordinates – on the likelihood of extra-group paternity (EGP) in offspring from dominant females in the Seychelles warbler (subset A).**

| **Fixed term** | **β** | **95% CI** | **ω_p_** |
| --- | --- | --- | --- |
| (Intercept) | -0.47 | -0.67, -0.27 | - |
| **Number of subordinates** | **0.27** | **0.09, 0.44** | **1.00** |
| Population breeding density | -0.11 | -0.31 - 0.09 | 0.73 |
| Pairwise relatedness | 0.06 | -0.12 - 0.25 | 0.48 |
| Territory quality | 0.009 | -0.08 - 0.10 | 0.21 |
| Number of helpers | - | - | - |
| Local breeding density | - | - | - |
| **Random term** | **σ²** | **95% CI** | **n** |
| Mother ID | 0.19 | 0.00, 0.88 | 313 |
| **Social male ID** | **0.55** | **0.27, 1.09** | **311** |
| Year | 0.00 | 0.00, 0.25 | 17 |
| **Response: dominant female EGP likelihood (n = 816 offspring).**  Candidate models: 64. Top set models: 6 (see Table S15 for details). | | | |

Full model-averaged estimates (β), 95% confidence intervals (CIs) and relative importance (ω_p_) are shown for all socio-ecological predictors featuring in the top model set (ΔAICc ≤ 2). Random effect variances (σ²) and their 95% CIs in the best model are also shown. Predictors whose CIs do not overlap with zero are given in bold.

**Table S5. Model-averaged parameters: the effect of socio-ecological predictors – including whether mature male and female subordinates are present – on the likelihood of extra-group paternity (EGP) in offspring from dominant females in the Seychelles warbler (subset A).**

| **Fixed term** | **β** | **95% CI** | **ω_p_** |
| --- | --- | --- | --- |
| (Intercept) | -0.67 | -0.91, -0.43 | - |
| **Male subordinate presence** | **0.60** | **0.15, 1.05** | **1.00** |
| **Female subordinate presence** | **0.40** | **0.001, 0.81** | **1.00** |
| Population breeding density | -0.12 | -0.32, 0.08 | 0.77 |
| Pairwise relatedness | 0.06 | -0.12, 0.20 | 0.43 |
| Territory quality | 0.01 | -0.08, 0.10 | 0.20 |
| Number of helpers | 0.003 | -0.06, 0.06 | 0.09 |
| Local breeding density | - | - | - |
| **Random term** | **σ²** | **95% CI** | **n** |
| Mother ID | 0.22 | 0.00, 0.91 | 313 |
| **Social male ID** | **0.52** | **0.24, 1.07** | **311** |
| Year | 0.00 | 0.00, 0.25 | 17 |
| **Response: dominant female EGP likelihood (n = 816 offspring).**  Candidate models: 128. Top set models: 7 (see Table S16 for details). | | | |

Full model-averaged estimates (β), 95% confidence intervals (CIs) and relative importance (ω_p_) are shown for all socio-ecological predictors featuring in the top model set (ΔAICc ≤ 2). Random effect variances (σ²) and their 95% CIs in the best model are also shown. Predictors whose CIs do not overlap with zero are given in bold.

**Table S6. Model-averaged parameters: the effect of socio-ecological predictors – including whether male and female helpers were present – on the likelihood of extra-group paternity (EGP) in offspring from dominant females in the Seychelles warbler (subset A).**

| **Fixed term** | **β** | **95% CI** | **ω_p_** |
| --- | --- | --- | --- |
| (Intercept) | -0.46 | -0.66, -0.26 | - |
| **Group size** | **0.35** | **0.17, 0.52** | **1.00** |
| Population breeding density | -0.06 | -0.23, 0.11 | 0.50 |
| Pairwise relatedness | 0.06 | -0.12, 0.25 | 0.49 |
| Male helper presence | -0.05 | -0.43, 0.33 | 0.23 |
| Territory quality | 0.01 | -0.08, 0.11 | 0.23 |
| Female helper presence | **-** | **-** | **-** |
| Local breeding density | **-** | **-** | **-** |
| **Random term** | **σ²** | **95% CI** | **n** |
| Mother ID | 0.15 | 0.00, 0.86 | 313 |
| **Social male ID** | **0.58** | **0.30, 1.10** | **311** |
| Year | 0.00 | 0.00, 0.25 | 17 |
| **Response: dominant female EGP likelihood (n = 816 offspring).**  Candidate models: 128. Top set models: 12 (see Table S17 for details). | | | |

Full model-averaged estimates (β), 95% confidence intervals (CIs) and relative importance (ω_p_) are shown for all socio-ecological predictors featuring in the top model set (ΔAICc ≤ 2). Random effect variances (σ²) and their 95% CIs in the best model are also shown. Predictors whose CIs do not overlap with zero are given in bold.

**Table S7. Model-averaged parameters: the effect of socio-ecological predictors – including group size – on the likelihood of extra-group paternity (EGP) in offspring from dominant females in the Seychelles warbler (subset B: with non-extrapolated territory quality data).**

| **Fixed term** | **β** | **95% CI** | **ω_p_** |
| --- | --- | --- | --- |
| (Intercept) | -0.48 | -0.68, -0.28 | - |
| **Group size** | **0.31** | **0.13, 0.49** | **1.00** |
| Population breeding density | -0.05 | -0.23, 0.12 | 0.42 |
| Pairwise relatedness | 0.03 | -0.11, 0.16 | 0.30 |
| Territory quality | 0.007 | -0.07, 0.08 | 0.13 |
| Number of helpers | - | - | - |
| Local breeding density | - | - | - |
| **Random term** | **σ²** | **95% CI** | **n** |
| Mother ID | 0.00 | 0.00, 0.79 | 287 |
| Social male ID | 0.00 | 0.00, 1.06 | 286 |
| Year | 0.00 | 0.00, 0.30 | 14 |
| **Response: dominant female EGP likelihood (n = 636 offspring).**  Candidate models: 64. Top set models: 5 (see Table S18 for details). | | | |

Full model-averaged estimates (β), 95% confidence intervals (CIs) and relative importance (ω_p_) are shown for all socio-ecological predictors featuring in the top model set (ΔAICc ≤ 2). Random effect variances (σ²) and their 95% CIs in the best model are also shown. Predictors whose CIs do not overlap with zero are given in bold.

**Table S8. Model-averaged parameters: the effect of socio-ecological predictors – including group size, breeding synchrony and clutch size – on the likelihood of extra-group paternity (EGP) in offspring from dominant mothers in the Seychelles warbler (subset C).**

| **Fixed term** | **β** | **95% CI** | **ω_p_** |
| --- | --- | --- | --- |
| (Intercept) | -0.31 | -0.55, -0.08 | - |
| **Group Size** | **0.40** | **0.14, 0.65** | **1.00** |
| Territory quality | 0.19 | -0.11, 0.49 | 0.79 |
| Population breeding density | -0.02 | -0.36, 0.15 | 0.18 |
| Number of helpers | -0.02 | -0.16, 0.12 | 0.11 |
| Pairwise relatedness | 0.01 | -0.10, 0.12 | 0.11 |
| Population breeding synchrony | -0.01 | -0.11, 0.09 | 0.10 |
| Local breeding synchrony | -0.006 | -0.09, 0.08 | 0.08 |
| Local breeding density | -0.004 | -0.08, 0.07 | 0.08 |
| Clutch size | - | - | - |
| Pop. breed. density x pop. breed. synchrony | - | - | - |
| Local breed. density x local breed. synchrony | **-** | **-** | **-** |
| **Random term** | **σ²** | **95% CI** | **n** |
| **Social pair ID** | **0.82** | **0.83, 0.91** | **250** |
| Year | 0.00 | 0.00, 0.38 | 17 |
| Nest ID | 0.00 | 0.00, 1.00 | 336 |
| **Response: dominant female EGP likelihood (n = 356 offspring).**  Candidate models: 800. Top set models: 9 (see Table S19 for details). | | | |

Full model-averaged estimates (β), 95% confidence intervals (CIs) and relative importance (ωp) are shown for all socio-ecological predictors featuring in the top model set (ΔAICc ≤ 2). Random effect variances (σ²) and their 95% CIs in the best model are also shown. An interaction between fixed effects is indicated by ‘x’. Predictors whose CIs do not overlap with zero are given in bold.

**Table S9. Model-averaged parameters: the effect of socio-ecological predictors – including male and female group size – on the likelihood of extra-group paternity (EGP) in offspring from subordinate females in the Seychelles warbler.**

| **Fixed term** | **β** | **95% CIs** | **ω_p_** |
| --- | --- | --- | --- |
| (Intercept) | 0.09 | -0.50, 0.67 | - |
| **Pairwise relatedness** | **0.71** | **0.08, 1.34** | **1.00** |
| Female group size | 0.43 | -0.33, 1.19 | 0.77 |
| Male group size | 0.24 | -0.44, 0.91 | 0.48 |
| Territory quality | 0.05 | -0.31, 0.41 | 0.19 |
| Number of helpers | -0.07 | -0.48, 0.34 | 0.16 |
| Local breeding density | -0.01 | -0.21, 0.18 | 0.07 |
| Population breeding density | - | - | - |
| **Random term** | **σ²** | **95% CI** | **n** |
| Social pair ID | 1.26 | 0.00, 2.98 | 60 |
| Year | 0.00 | 0.00, 0.90 | 16 |
| **Response: subordinate female EGP likelihood (n = 101 offspring).**  Candidate models: 128. Top set models: 9 (see Table S21 for details). | | | |

Full model-averaged estimates (β), 95% confidence intervals (CIs) and relative importance (ω_p_) are shown for all socio-ecological predictors featuring in the top model set (ΔAICc ≤ 2). Random effect variances (σ²) and their 95% CIs in the best model are also shown. Predictors whose CIs do not overlap with zero are given in bold.

**Table S10. Model-averaged parameters: the effect of socio-ecological predictors – including the number of mature subordinates – on the likelihood of extra-group paternity (EGP) in offspring from subordinate females in the Seychelles warbler.**

| **Fixed term** | **β** | **95% CIs** | **ω_p_** |
| --- | --- | --- | --- |
| (Intercept) | 0.09 | -0.45, 0.64 | - |
| Pairwise relatedness | 0.63 | -0.07, 1.18 | 1.00 |
| Number of subordinates | 0.49 | -0.31, 1.28 | 0.81 |
| Territory quality | 0.05 | -0.30, 0.69 | 0.17 |
| Local breeding density | -0.03 | -0.29, 0.23 | 0.14 |
| Number of helpers | -0.03 | -0.31, 0.25 | 0.14 |
| Population breeding density | - | - | - |
| **Random term** | **σ²** | **95% CI** | **n** |
| Mother ID | 1.40 | 0.00, 2.11 | 53 |
| Social male ID | 0.00 | 0.00, 2.70 | 58 |
| Year | 0.00 | 0.00, 0.90 | 16 |
| **Response: subordinate female EGP likelihood (n = 101 offspring).**  Candidate models: 64. Top set models: 5 (see Table S22 for details). | | | |

Full model-averaged estimates (β), 95% confidence intervals (CIs) and relative importance (ω_p_) are shown for all socio-ecological predictors featuring in the top model set (ΔAICc ≤ 2). Random effect variances (σ²) and their 95% CIs in the best model are also shown.

**Table S11. Model-averaged parameters: the effect of socio-ecological predictors – including whether mature male and female subordinates are present – on the likelihood of extra-group paternity (EGP) in offspring from subordinate females in the Seychelles warbler.**

| **Fixed term** | **β** | **95% CIs** | **ω_p_** |
| --- | --- | --- | --- |
| (Intercept) | -0.16 | -0.80, 0.49 | - |
| Female subordinate presence | 1.03 | -0.53, 2.59 | 0.88 |
| Pairwise relatedness | 0.49 | -0.15, 1.13 | 0.86 |
| Local breeding density | -0.03 | -0.29, 0.22 | 0.13 |
| Territory quality | 0.03 | -0.24, 0.30 | 0.12 |
| Male subordinate presence | 0.07 | -0.56, 0.70 | 0.11 |
| Number of helpers | -0.03 | -0.26, 0.21 | 0.11 |
| Population breeding density | - | - | - |
| **Random term** | **σ²** | **95% CI** | **n** |
| Social Pair ID | 0.97 | 0.00, 2.54 | 60 |
| Year | 0.00 | 0.00, 0.92 | 16 |
| **Response: subordinate female EGP likelihood (n = 101 offspring).**  Candidate models: 128. Top set models: 7 (see Table S23 for details).  Full model-averaged estimates (β), 95% confidence intervals (CIs) and relative importance (ω_p_) are shown for all socio-ecological predictors featuring in the top model set (ΔAICc ≤ 2). Random effect variances (σ²) and their 95% CIs in the best model are also shown. | | | |

**Table S12. Model-averaged parameters: the effect of socio-ecological predictors – including whether male and female helpers are present – on the likelihood of extra-group paternity (EGP) in offspring from subordinate females in the Seychelles warbler.**

| **Fixed term** | **β** | **95% CIs** | **ω_p_** |
| --- | --- | --- | --- |
| (Intercept) | 0.16 | -0.42, 0.75 | - |
| Group size | 0.64 | -0.06, 1.35 | 1.00 |
| **Pairwise relatedness** | **0.67** | **0.06, 1.28** | **1.00** |
| Female helper presence | -0.25 | -1.49, 0.99 | 0.25 |
| Local breeding density | -0.04 | -0.33, 0.25 | 0.18 |
| Territory quality | 0.03 | -0.27, 0.34 | 0.16 |
| Population breeding density | - | - | - |
| Male helper presence | - | - | - |
| **Random term** | **σ²** | **95% CI** | **n** |
| Social pair ID | 1.16 | 0.00, 2.72 | 60 |
| Year | 0.00 | 0.00, 0.94 | 16 |
| **Response: subordinate female EGP likelihood (n = 101 offspring).**  Candidate models: 128. Top set models: 4 (see Table S24 for details). | | | |

Full model-averaged estimates (β), 95% confidence intervals (CIs) and relative importance (ω_p_) are shown for all socio-ecological predictors featuring in the top model set (ΔAICc ≤ 2). Random effect variances (σ²) and their 95% CIs in the best model are also shown. Predictors whose CIs do not overlap with zero are given in bold.

**Table S13. Top model set: analysis of the effect of socio-ecological predictors – including group size – on the likelihood of extra-group paternity (EGP) in offspring from dominant females in the Seychelles warbler (subset A).**

| **Analysis** | **Model rank** | **Model** | **AICc** | **ΔAICc** | **ω_m_** |
| --- | --- | --- | --- | --- | --- |
| Dominant female | 1 | GS + PBD | 1069.91 | 0.00 | 0.15 |
| EGP likelihood | 2 | GS + PBD + R | 1070.00 | 0.09 | 0.14 |
|  | 3 | GS | 1070.00 | 0.10 | 0.14 |
|  | 4 | GS *+ R* | 1070.14 | 0.23 | 0.13 |
|  | 5 | GS + H + PBD | 1071.53 | 1.62 | 0.07 |
|  | 6 | GS + TQ | 1071.53 | 1.62 | 0.07 |
|  | 7 | GS *+ R* + TQ | 1071.60 | 1.70 | 0.06 |
|  | 8 | GS + H | 1071.69 | 1.78 | 0.06 |
|  | 9 | GS + PBD + TQ | 1071.78 | 1.88 | 0.06 |
|  | 10 | GS + H + PBD + R | 1071.79 | 1.88 | 0.06 |
|  | 11 | GS + PBD *+ R* + TQ | 1071.84 | 1.94 | 0.06 |

Group size, GS; population breeding density, PBD; relatedness*, R*; number of helpers, H; territory quality, TQ.

For each model included in the top set (ΔAICc ≤ 2) the table shows rank, fixed predictors, AICC, ΔAICc and model weight (ω_m_).

**Table S14. Top model set: analysis of the effect of socio-ecological predictors – including male and female group size – on the likelihood of extra-group paternity (EGP) in offspring from dominant females in the Seychelles warbler (subset A).**

| **Analysis** | **Model rank** | **Model** | **AICc** | **ΔAICc** | **ω_m_** |
| --- | --- | --- | --- | --- | --- |
| Dominant female | 1 | FGS + MGS + PBD *+ R* | 1070.91 | 0.00 | 0.20 |
| EGP likelihood | 2 | FGS + MGS + PBD | 1071.00 | 0.10 | 0.19 |
|  | 3 | FGS + MGS *+ R* | 1071.81 | 0.91 | 0.13 |
|  | 4 | FGS + MGS | 1071.83 | 0.93 | 0.13 |
|  | 5 | FGS + MGS + H + PBD | 1072.48 | 1.58 | 0.09 |
|  | 6 | FGS + MGS + H + PBD + R | 1072.55 | 1.64 | 0.09 |
|  | 7 | FGS + MGS + PBD *+ R* + TQ | 1072.59 | 1.69 | 0.09 |
|  | 8 | FGS + MGS + PBD *+* TQ | 1072.74 | 1.83 | 0.08 |

Female group size, FGS; male group size, MGS; population breeding density, PBD; relatedness*, R*; number of helpers, H; territory quality, TQ.

For each model included in the top set (ΔAICc ≤ 2) the table shows rank, fixed predictors, AICC, ΔAICc and model weight (ω_m_).

**Table S15. Top model set: analysis of the effect of socio-ecological predictors – including the number of mature subordinates – on the likelihood of extra-group paternity (EGP) in offspring from dominant females in the Seychelles warbler (subset A).**

| **Analysis** | **Model rank** | **Model** | **AICc** | **ΔAICc** | **ω_m_** |
| --- | --- | --- | --- | --- | --- |
| Dominant female | 1 | Sub + PBD | 1076.31 | 0.00 | 0.27 |
| EGP likelihood | 2 | Sub + PBD *+ R* | 1076.43 | 0.12 | 0.25 |
|  | 3 | Sub | 1077.55 | 1.24 | 0.14 |
|  | 4 | Sub *+ R* | 1077.74 | 1.43 | 0.13 |
|  | 5 | Sub + PBD + TQ | 1078.13 | 1.82 | 0.11 |
|  | 6 | Sub + PBD + *R* + TQ | 1078.22 | 1.91 | 0.10 |

Number of mature subordinates, Sub; population breeding density, PBD; relatedness*, R*; number of helpers, H; territory quality, TQ.

For each model included in the top set (ΔAICc ≤ 2) the table shows rank, fixed predictors, AICC, ΔAICc and model weight (ω_m_).

**Table S16. Top model set: analysis of the effect of socio-ecological predictors – including mature subordinate presence split by sex – on the likelihood of extra-group paternity (EGP) in offspring from dominant females in the Seychelles warbler (subset A).**

| **Analysis** | **Model rank** | **Model** | **AICc** | **ΔAICc** | **ω_m_** |
| --- | --- | --- | --- | --- | --- |
| Dominant female | 1 | Fsub + Msub + PBD | 1077.13 | 0.00 | 0.25 |
| EGP likelihood | 2 | Fsub + Msub + PBD *+ R* | 1077.30 | 0.17 | 0.23 |
|  | 3 | Fsub + Msub | 1078.52 | 1.39 | 0.12 |
|  | 4 | Fsub + Msub *+ R* | 1078.76 | 1.63 | 0.11 |
|  | 5 | Fsub + Msub + PBD + TQ | 1078.89 | 1.75 | 0.10 |
|  | 6 | Fsub + Msub + PBD + TQ *+ R* | 1079.01 | 1.88 | 0.10 |
|  | 7 | Fsub + Msub + H + PDB | 1079.09 | 1.96 | 0.09 |

Presence of mature female subordinates, Fsub; presence of mature male subordinates, Msub; population breeding density, PBD; relatedness*, R*; number of helpers, H; territory quality, TQ.

For each model included in the top set (ΔAICc ≤ 2) the table shows rank, fixed predictors, AICC, ΔAICc and model weight (ω_m_).

**Table S17. Top model set: analysis of the effect of socio-ecological predictors – with helper presence split by sex – on the likelihood of extra-group paternity (EGP) in offspring from dominant females in the Seychelles warbler (subset A).**

| **Analysis** | **Model rank** | **Model** | **AICc** | **ΔAICc** | **ω_m_** |
| --- | --- | --- | --- | --- | --- |
| Dominant female | 1 | GS + PBD | 1069.91 | 0.00 | 0.14 |
| EGP likelihood | 2 | GS + PBD + *R* | 1070.00 | 0.09 | 0.13 |
|  | 3 | GS | 1070.00 | 0.10 | 0.13 |
|  | 4 | GS *+ R* | 1070.14 | 0.23 | 0.13 |
|  | 5 | GS + TQ | 1071.53 | 1.62 | 0.06 |
|  | 6 | GS + MH + PBD | 1071.60 | 1.69 | 0.06 |
|  | 7 | GS *+ R* + TQ | 1071.60 | 1.70 | 0.06 |
|  | 8 | GS + MH | 1071.63 | 1.72 | 0.06 |
|  | 9 | GS *+*MH + PBD + *R* | 1071.67 | 1.77 | 0.06 |
|  | 10 | GS + MH + *R* | 1071.74 | 1.83 | 0.06 |
|  | 11 | GS + PBD *+* TQ | 1071.78 | 1.88 | 0.06 |
|  | 12 | GS + PBD + *R* + TQ | 1071.84 | 1.94 | 0.05 |

Group size, GS; population breeding density, PBD; relatedness*, R*; territory quality, TQ; presence of male helpers, MH.

For each model included in the top set (ΔAICc ≤ 2) the table shows rank, fixed predictors, AICC, ΔAICc and model weight (ω_m_).

**Table S18. Top model set: analysis of the effect of socio-ecological predictors – including group size – on the likelihood of extra-group paternity (EGP) in offspring from dominant females in the Seychelles warbler (subset B).**

| **Analysis** | **Model rank** | **Model** | **AICc** | **ΔAICc** | **ω_m_** |
| --- | --- | --- | --- | --- | --- |
| Dominant female | 1 | GS | 842.04 | 0.00 | 0.30 |
| EGP likelihood | 2 | GS + PBD | 842.20 | 0.16 | 0.27 |
|  | 3 | GS *+ R* | 843.34 | 1.30 | 0.15 |
|  | 4 | GS + PDB *+ R* | 843.41 | 1.37 | 0.15 |
|  | 5 | GS + TQ | 843.73 | 1.68 | 0.13 |

Group size, GS; population breeding density, PBD; relatedness*, R*; territory quality, TQ.

For each model included in the top set (ΔAICc ≤ 2) the table shows rank, fixed predictors, AICC, ΔAICc and model weight (ω_m_).

**Table S19. Top model set: analysis of the effect of socio-ecological predictors – including group size – on the likelihood of extra-group paternity (EGP) in offspring from dominant females in the Seychelles warbler (subset C).**

| **Analysis** | **Model rank** | **Model** | **AICc** | **ΔAICc** | **ω_m_** |
| --- | --- | --- | --- | --- | --- |
| Dominant female | 1 | GS + TQ | 482.83 | 0.00 | 0.21 |
| EGP likelihood | 2 | GS | 483.86 | 1.03 | 0.13 |
|  | 3 | GS + H + TQ | 484.15 | 1.32 | 0.11 |
|  | 4 | GS + *R* + TQ | 484.19 | 1.36 | 0.11 |
|  | 5 | GS + PBS + TQ | 484.32 | 1.49 | 0.10 |
|  | 6 | GS *+* PBD + TQ | 484.38 | 1.56 | 0.10 |
|  | 7 | GS + PBD | 484.61 | 1.79 | 0.09 |
|  | 8 | GS + LBS + TQ | 484.65 | 1.83 | 0.08 |
|  | 9 | GS + LBD | 484.77 | 1.94 | 0.08 |

Group size, GS; territory quality, TQ; umber of helpers, H; relatedness*, R*; population breeding synchrony, PBS; population breeding density, PBD; local breeding synchrony, LBS; local breeding density, LBD.

For each model included in the top set (ΔAICc ≤ 2) the table shows rank, fixed predictors, AICC, ΔAICc and model weight (ω_m_).

**Table S20. Top model set: analysis of the effect of socio-ecological predictors – including group size – on the likelihood of extra-group paternity (EGP) in offspring from subordinate females in the Seychelles warbler.**

| **Analysis** | **Model rank** | **Model** | **AICc** | **ΔAICc** | **ω_m_** |
| --- | --- | --- | --- | --- | --- |
| Subordinate female | 1 | GS *+ R* | 136.55 | 0.00 | 0.51 |
| EGP likelihood | 2 | GS + H *+ R* | 137.75 | 1.20 | 0.28 |
|  | 3 | GS *+ R* + TQ | 138.31 | 1.76 | 0.21 |

Group size, GS; relatedness*, R*; number of helpers, H; territory quality, TQ.

For each model included in the top set (ΔAICc ≤2) the table shows rank, fixed predictors, AICC, ΔAICc and model weight (ω_m_).

**Table S21. Top model set: analysis of the effect of socio-ecological predictors – including male and female group size – on the likelihood of extra-group paternity (EGP) in offspring from subordinate females in the Seychelles warbler.**

| **Analysis** | **Model rank** | **Model** | **AICc** | **ΔAICc** | **ω_m_** |
| --- | --- | --- | --- | --- | --- |
| Subordinate female | 1 | FGS *+ R* | 137.99 | 0.00 | 0.18 |
| EGP likelihood | 2 | FGS + MGS *+ R* | 138.18 | 0.19 | 0.16 |
|  | 3 | MGS *+ R* | 138.75 | 0.76 | 0.12 |
|  | 4 | FGS + MGS *+* H + TQ | 138.93 | 0.94 | 0.11 |
|  | 5 | R | 139.00 | 1.01 | 0.11 |
|  | 6 | FGS *+ R* + TQ | 139.44 | 1.45 | 0.09 |
|  | 7 | FGS + MGS *+ R* + TQ | 139.72 | 1.73 | 0.08 |
|  | 8 | FGS + H *+ R* | 139.73 | 1.75 | 0.08 |
|  | 9 | FGS + LBD *+ R* | 139.88 | 1.90 | 0.07 |

Female group size, FGS; male groups size, MGS; relatedness*, R*; territory quality, TQ; number of helpers, H.

For each model included in the top set (ΔAICc ≤ 2) the table shows rank, fixed predictors, AICC, ΔAICc and model weight (ω_m_).

**Table S22. Top model set: analysis of the effect of socio-ecological predictors – including the number of mature subordinates – on the likelihood of extra-group paternity (EGP) in offspring from subordinate females in the Seychelles warbler.**

| **Analysis** | **Model rank** | **Model** | **AICc** | **ΔAICc** | **ω_m_** |
| --- | --- | --- | --- | --- | --- |
| Subordinate female | 1 | Sub *+ R* | 137.72 | 0.00 | 0.36 |
| EGP likelihood | 2 | *R* | 139.00 | 1.28 | 0.19 |
|  | 3 | Sub + *R* + TQ | 139.29 | 1.57 | 0.17 |
|  | 4 | H + Sub + *R* | 139.61 | 1.89 | 0.14 |
|  | 5 | LBD + Sub + *R* | 139.67 | 1.95 | 0.14 |

Number of mature subordinates, Sub; relatedness*, R*; territory quality, TQ; number of helpers, H; local breeding density, LBD.

For each model included in the top set (ΔAICc ≤ 2) the table shows rank, fixed predictors, AICC, ΔAICc and model weight (ω_m_).

**Table S23. Top model set: analysis of the effect of socio-ecological predictors – including whether mature male and female subordinates were present – on the likelihood of extra-group paternity (EGP) in offspring from subordinate females in the Seychelles warbler.**

| **Analysis** | **Model rank** | **Model** | **AICc** | **ΔAICc** | **ω_m_** |
| --- | --- | --- | --- | --- | --- |
| Subordinate female | 1 | Fsub *+ R* | 137.84 | 0.00 | 0.26 |
| EGP likelihood | 2 | *R* | 139.04 | 1.20 | 0.14 |
|  | 3 | Fsub + LBD *+ R* | 139.25 | 1.41 | 0.13 |
|  | 4 | Fsub | 139.35 | 1.50 | 0.12 |
|  | 5 | Fsub *+ R* + TQ | 139.41 | 1.56 | 0.12 |
|  | 6 | Fsub + H + *R* | 139.48 | 1.64 | 0.11 |
|  | 7 | Fsub + Msub *+ R* | 139.51 | 1.67 | 0.11 |

Presence of mature female subordinates, Fsub; relatedness*, R*; local breeding density, LBD; territory quality, TQ; presence of mature male subordinates, Msub.

For each model included in the top set (ΔAICc ≤ 2) the table shows rank, fixed predictors, AICC, ΔAICc and model weight (ω_m_).

**Table S24. Top model set: analysis of the effect of socio-ecological predictors – including whether male and female helpers were present – on the likelihood of extra-group paternity (EGP) in offspring from subordinate females in the Seychelles warbler.**

| **Analysis** | **Model rank** | **Model** | **AICc** | **ΔAICc** | **ω_m_** |
| --- | --- | --- | --- | --- | --- |
| Subordinate female | 1 | GS *+ R* | 136.76 | 0.00 | 0.41 |
| EGP likelihood | 2 | FH + GS *+ R* | 137.79 | 1.02 | 0.25 |
|  | 3 | GS + LBD *+ R* | 138.40 | 1.64 | 0.18 |
|  | 4 | GS + *R* + TQ | 138.64 | 1.88 | 0.16 |

Group size, GS; relatedness*, R*; female helper presence, FH; local breeding density, LBD; territory quality, TQ.

For each model included in the top set (ΔAICc ≤ 2) the table shows rank, fixed predictors, AICC, ΔAICc and model weight (ω_m_).
